# Mapping three-dimensional surface deformation caused by the 2010 Haiti earthquake using advanced satellite radar interferometry

**DOI:** 10.1371/journal.pone.0188286

**Published:** 2017-11-16

**Authors:** Hyung-Sup Jung, Soo-Min Hong

**Affiliations:** 1 Department of Geoinformatics, The University of Seoul, Dongdaemun-gu, Seoul, Korea; 2 Department of English Language and Literature, The University of Seoul, Dongdaemun-gu, Seoul, Korea; Universidade de Aveiro, PORTUGAL

## Abstract

Mapping three-dimensional (3D) surface deformation caused by an earthquake is very important for the environmental, cultural, economic and social sustainability of human beings. Synthetic aperture radar (SAR) systems made it possible to measure precise 3D deformations by combining SAR interferometry (InSAR) and multiple aperture interferometry (MAI). In this paper, we retrieve the 3D surface deformation field of the 2010 Haiti earthquake which occurred on January 12, 2010 by a magnitude 7.0 Mw by using the advanced interferometric technique that integrates InSAR and MAI data. The surface deformation has been observed by previous researchers using the InSAR and GPS method, but 3D deformation has not been measured yet due to low interferometric coherence. The combination of InSAR and MAI were applied to the ALOS PALSAR ascending and descending pairs, and were validated with the GPS in-situ measurements. The archived measurement accuracy was as little as 1.85, 5.49 and 3.08 cm in the east, north and up directions, respectively. This result indicates that the InSAR/MAI-derived 3D deformations are well matched with the GPS deformations. The 3D deformations are expected to allow us to improve estimation of the area affected by the 2010 Haiti earthquake.

## Introduction

For advances in the geological interpretation of geohazards such as earthquakes and volcanic eruptions, three-dimensional (3D) surface deformation mapping has been becoming increasingly more essential. Synthetic aperture radar (SAR) systems enable us to measure 3D surface deformations precisely by combining SAR interferometry (InSAR) and multiple aperture interferometry (MAI) [1–14]. The InSAR method has been successful in mapping ground surface deformations in the line-of-sight (LOS) direction over areas of thousands of square kilometers. However, InSAR has a limitation that it cannot measure the along-track deformation, which is approximate to the deformation in the North-South direction because most SAR satellites have near-polar orbits [2,4–6]. Since the InSAR method has a difficulty in measuring along-track deformations, the MAI method is required to retrieve the three-dimensional deformation. MAI is implemented by split-beam InSAR processing. The main idea has been proposed by [15] and further improvements has been done by [16–19]. The detail processing steps of this method include 1) the creation of forward- and backward-looking differential interferograms using azimuth split-band filtering and 2) the construction of an MAI interferogram from the two interferograms. Moreover, this method has been applied to estimate ionospheric phase screen from an MAI interferogram [20–22]. The 3D deformation measurement accuracies obtained from the InSAR and MAI combination were about 1.6, 3.6 and 2.1 cm for L-band ALOS PALSAR data in the east, north and up directions, respectively [2], and about 0.86, 1.04 and 0.55 cm for X-band COSMO-SkyMed data in the east, north and up directions, respectively [6]. Currently, the state-of-the-art technique for the 3D deformation measurement allows us to measure a several centimeter deformation.

The 2010 magnitude 7.0 Mw Haiti earthquake occurred on January 12, 2010. Since its epicenter was located as close as about 25 km south west of Port-au-Prince, which is Haiti’s capital, an estimated three million people were affected and notable landmark buildings were significantly damaged by the earthquake [23]. It was reported that about 250,000 residences and 30,000 commercial buildings were severely broken. Notably, most buildings (approximately 80–90%) in the city of Leogane near the epicenter of the earthquake were severely damaged or destroyed [23]. The government of Haiti estimated that more than 300,000 persons were dead or missing, about 300,000 persons were injured, and over 1,300,000 persons were homeless [23]. Overall losses from the Haiti earthquake reaches approximately US$ 14,000,000,000 [24]. The 2010 Haiti earthquake gives us a lesson that measuring and monitoring earthquakes is very important for a threefold purpose: the environmental, cultural, economic, and social sustainability of human beings.

The surface deformation caused by the 2010 Haiti earthquake has been observed by InSAR and GPS methods [25– 28]. The fan-delta uplift and mountain subsidence during the 2010 Haiti earthquake has been observed by the InSAR method [25] and the sudden uplift of the delta by 60–80 cm after the earthquake was measured by the InSAR method [26]. Ground uplift in a coastal area was measured by both InSAR and GPS methods [27]. Based on the co-seismic observations, a simple rupture geometry with a single fault made of two subsegments has also been proposed by [27] and a more complex geometry with multiple faults has been proposed by [28]. The proposed fault geometries, which were calculated by using the finite fault model, have been compared through dynamic rupture simulations [29].

Although it can be very important to measure 3D deformation field to improve the finite fault model accuracy, the 3D ground deformation was not retrieved from these previous studies. The reasons are as following: 1) the study area is a highly mountainous region with steep mountains and narrow valleys, 2) the data pairs used for this study have relatively long time intervals. Thus, the geometric distortion of the interferometric pairs is very high due to the SAR imaging geometry and the interferometric coherence is low as well. It all means that it is very hard to apply the InSAR and MAI techniques to the Haiti interferometric pairs. Particularly, the MAI method can have a difficulty in measuring the along-track deformation because it requires higher coherence than the InSAR method.

In this paper, we three-dimensionally measure the ground surface deformation caused by the 2010 Haiti earthquake using the InSAR and MAI methods and validate the achieved accuracy of the 3D deformations from the GPS measurements. The data used for this study is the ALOS PALSAR ascending pair of FBS (fine beam single polarization) acquisitions on February 9, 2008 and February 14, 2010, and the descending pairs of FBS acquisitions on March 9, 2009 and January 25, 2010. The interferometric pairs are exactly the same to the pairs in the previous studies [27–29]. In addition, the severe topographic distortion of the MAI measurement is shown and corrected. We expect that the 3D measurement can give us to a better understanding of the fault’s behavior of the 2010 Haiti earthquake.

## Study area and datasets

**Fig 1:**
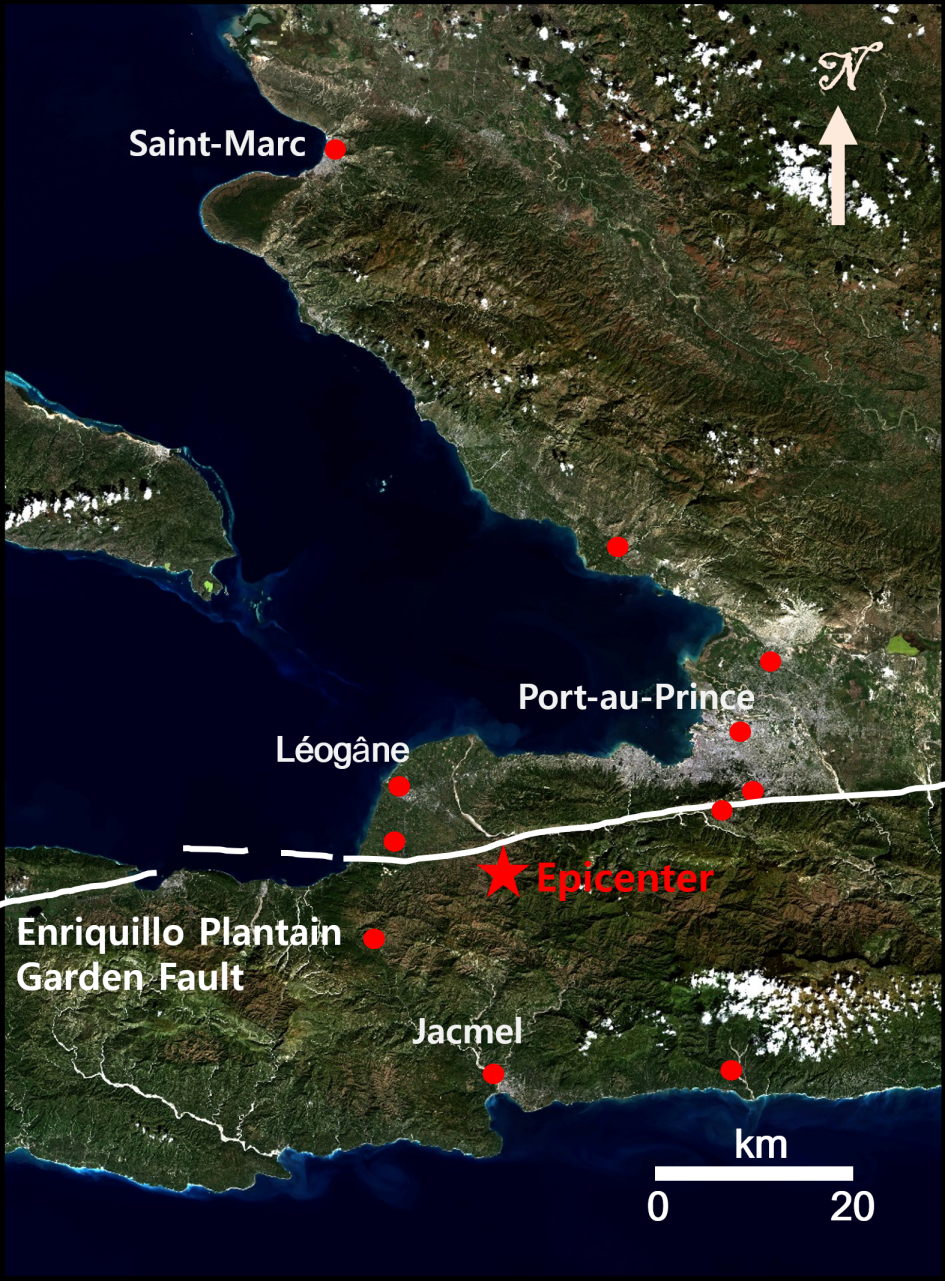
Study area. The small red solid circles indicate the positions of GPS stations, and the red star represents the epicenter of the 2010 Haiti earthquake.

On January 12, 2010, faults associated with the Enriquillo-Plantain Garden left-lateral strike-slip fault zone (EPGFZ) in the Caribbean-North America plate boundary were struck by an Mw 7.0 earthquake [30]. The seismic event has caused severe damages as follows: 1) people were killed and injured and 2) social, economic, and environmental sustainability in the development of Haiti took a big backward step. Haiti is located in the island of Hispaniola and shares the island with the Dominican Republic. Haiti is a highly mountainous country, and hence the areas of sediment and floodplains were relatively rare in Haiti. Fig 1 shows the portion of Haiti that was struck by the 2010 Haiti earthquake. The epicenter of the Mw 7.0 earthquake is shown as the red star in Fig 1. The epicenter is very close (approximately 25 km) to the capital of Haiti, Port-au-Prince, and hence well-known landmark buildings were significantly damaged. Even though the surface deformation associated with the 2010 Haiti earthquake was very large, the significant surface rupture was not found [30]. Thus, Prentice et al. [30] have reported that the Enriquillo-Plantain Garden fault remains a significant seismic hazard.

The ALOS PALSAR ascending pairs of FBS (fine beam single polarization) acquired on February 9, 2008 and February 14, 2010 and the descending pairs of FBS acquired on March 9, 2009 and January 25, 2010 were used for retrieving 3D deformations by using the InSAR and MAI combination. The interferometric pairs have relatively long time intervals, which are about 2 year for ascending pair and about 1 year for descending pairs. It seems that the long time interval of InSAR pairs and the steep topography of Haiti make the precise 3D ground deformations retrieval from the InSAR and MAI methods very difficult. The GPS measurements estimated by [27] were used for the validation of the 3D deformations derived from InSAR and MAI and one-arc second SRTM DEM was used for the topographic correction of the InSAR and MAI processing. The GPS stations are shown as red solid circles in Fig 1.

## Methodology

The InSAR measurement has been widely used for mapping ground deformations, but the MAI measurement was not always applied because of the drawbacks that requires high coherence and complicate processing procedures. Recently, the MAI performance has been notably improved by reducing the interferometric phase errors and correcting the phase distortions [16–19]. Thus, the technical advances enable the MAI method to be applied to the 2010 Haiti co-seismic interferometric pairs. In this section, we will sketch out the advanced MAI method for ALOS PALSAR interferometry.

### MAI method

The MAI method utilizes a sub-aperture processing technique to create two images with slightly different line-of-sight (LOS) vectors in the azimuth direction: One image is a forward-looking image and the other image is a backward-looking image. Thus, the interferometry between master and slave forward-looking images is defined as the forward-looking SAR interferometry and the interferometry between master and slave backward-looking images is denoted as the backward-looking SAR interferometry. The difference between the forward- and backward-looking InSAR is MAI, which represents the along-track deformation. The MAI phase (*ϕ*_*MAI*_) can be defined as follows:

$$\phi_{MAI}=-\frac{4\pi }{l}nx,$$ (1)

where *x* is the along-track surface deformation, *l* is the effective antenna length (8.9m for ALOS PALSAR), and *n* is a normalized squint that is a fraction of the full aperture width [15]. If *n* = 0.5 is used for the MAI processing, *l* = 8.9 m for ALOS satellites and hence one fringe yields 8.9 m. When compared to the one fringe of about 11.8 cm in the InSAR processing, the measurement accuracy of MAI is much lower than that of InSAR. Suppose that a NS (North-South) strike-slip fault is formed by an earthquake, then we cannot measure the NS deformation by using the InSAR method. However, the MAI method enables us to measure the NS deformation. Therefore, the MAI measurement is very important to understand the surface deformation three-dimensionally despite the fact that the MAI measurement accuracy is much lower than the InSAR.

### Three-dimensional deformation measurement

The 3D components of the ground surface deformation can be retrieved by combining the InSAR and MAI measurements from ascending and descending interferometric pairs. The deformation vector (r) measured by using InSAR and MAI ascending and descending pairs can be defined by [2,31]:

r=U⋅d, (2)

where r can be defined as:

$$r={\left[\begin{array}{cccc} r_{InSAR,a} & r_{InSAR,d} & r_{MAI,a} & r_{MAI,d} \end{array}\right]}^{T},$$ (3)

where *r*_*InSAR*,*a*_ and *r*_*InSAR*,*d*_ are the InSAR measurements in the ascending and descending pairs, respectively, *r*_*MAI*,*a*_ and *r*_*MAI*,*d*_ are the MAI measurements in the ascending and descending pairs, respectively. *d* is 3D ground surface deformation as given by:

$$d={\left[\begin{array}{ccc} d_{east} & d_{north} & d_{up} \end{array}\right]}^{T},$$ (4)

where *d*_*east*_, *d*_*north*_ and *d*_*up*_ are the east, north and up components of the ground surface deformation, respectively. *U* is defined as:

$$U={\left[\begin{array}{cccc} u_{InSAR,a} & u_{InSAR,d} & u_{MAI,a} & u_{MAI,d} \end{array}\right]}^{T},$$ (5)

where ***u***_*InSAR*,*a*_ is the unit InSAR vector in the ascending pair as given by

$$u_{InSAR,a}={\left[\begin{array}{ccc} \text{sin}\theta_{a}\cdot \text{cos}\varphi_{a} & -\text{sin}\theta_{a}\cdot \text{sin}\varphi_{a} & -\text{cos}\theta_{a} \end{array}\right]}^{T},$$ (6)

where *θ*_***a***_ and *φ*_***a***_ are the radar incidence angle from vertical and the satellite track angle from north in the ascending pair, respectively, ***u***_*InSAR*,*d*_ is the descending unit InSAR vector and can be defined by replacing the subscript ‘a’ with ‘d’, ***u***_*MAI*,*a*_ is the unit MAI vector in the ascending pair as defined

$$u_{MAI,a}={\left[\begin{array}{ccc} \text{sin}\theta_{a} & \cdot \text{cos}\varphi_{a} & 0 \end{array}\right]}^{T},$$ (7)

and ***u***_*MAI*,*d*_ is the descending unit MAI vector which is defined by replacing the subscript ‘*a*’ with ‘*d*’ in Eq (7).

Finally, the 3D ground surface deformation ***d*** can be retrieved by using

$$d={\left(U^{T}\cdot W\cdot U\right)}^{-1}\cdot \left(U^{T}\cdot W\cdot r\right),$$ (8)

where *W* is the weighting matrix, which can be estimated from the standard deviations in the InSAR and MAI measurements [2, 31]. To retrieve 3D deformations, it is optimal to use the four measurements in Eq (3), one of the two MAI measurements cannot be used because they have a similar geometry.

## Results and discussion

**Fig 2:**
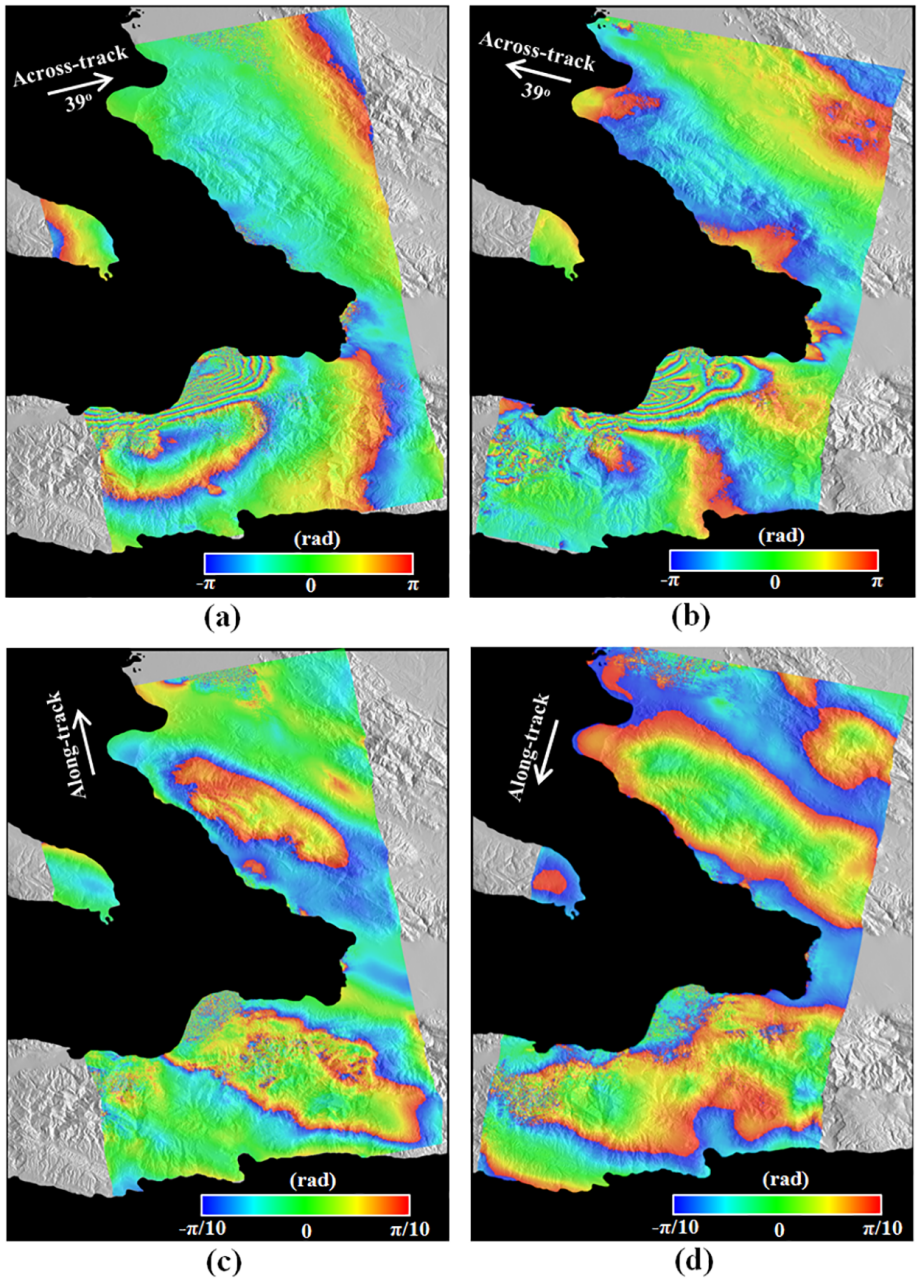
InSAR and MAI interferograms generated from (a) and (c) ALOS PALSAR ascending pairs acquired on February 9, 2008 and February 14, 2010, and (b) and (d) ALOS PALSAR descending pairs acquired on March 9, 2009 and January 25, 2010. It is noted that no topographic correction was applied to the MAI interferograms of (c) and (d).

To observe the surface deformation caused by the 2010 Haiti earthquake, the InSAR and MAI methods were applied to the ascending and descending ALOS PALSAR interferometric pairs. Fig 2(a) and 2(b) show the InSAR interferograms generated from the ascending pair acquired on February 9, 2008 and February 14, 2010 and the descending pair acquired on March 9, 2009 and January 25, 2010, respectively. The characteristics of ALOS PALSAR interferometric pairs used in this study are listed in Table 1.

**Table 1:** Characteristics of ALOS PALSAR interferometric pairs used in this study.

Pairs	Acquisition Date	Beam Mode	Inc. Angle (deg.)	*f*_*DC*,*f*_^c^ (Hz)	*f*_*DC*,*c*_^c^ (Hz)	*f*_*DC*,*b*_^c^ (Hz)	*Δf*_*D*,*S*_^d^ (Hz)	*B*_*p*_^e^ (m)	*ΔB*_*p*_^e^ (m)
Asc.	09/02/2008^a^ 14/02/2010	FBS^b^	38.7	480.6	76.5	-327.5	801.7	-417	0.15
Dsc.	09/03/2009^a^ 25/01/2010	FBS^b^	38.7	333.4	-70.7	-474.7	794.8	807	0.27

^a^Master image.

^b^Fine beam single polarizations.

^c^Forward, average, and backward Doppler centroids.

^d^Sub-aperture processing bandwidth.

^e^Perpendicular baseline and perpendicular baseline difference between forward- and backward-looking interferograms.

The ascending and descending InSAR interferograms have been produced by a complex multi-look operation of 8 looks in the range direction and 16 looks in the azimuth direction, which corresponds to about 60 and 50 m in range and azimuth directions, respectively. The multi-look operation has been applied by using a two-step strategy: 1) 2x4 multi-looking in the range and azimuth before the correction of the flat-Earth and topographic phase distortions and 2) 4x4 multi-looking after the correction to reduce interferometric phase noise efficiently [16]. Then the post-filtering of InSAR interferograms has been carried out by using the Goldstein adaptive filter [32] with a window size of 64 to reduce interferometric phase noise. As seen in Fig 2(a) and 2(b), because the epicenter of the 2010 Haiti earthquake is approximately 15 km south of the town of Léogâne, clear dense fringe patterns are presented in both the ascending and descending InSAR interferograms [28,29,33]. It indicates that large deformations occurs in the town of Léogâne. The different fringe pattern between the ascending and descending InSAR interferograms is due to their different viewing geometry. In the left-bottom of the descending interferogram (Red Arrow in Fig 2(b)), we can recognize that massive landslides occurred near to the city of Jacmel.

**Fig 3:**
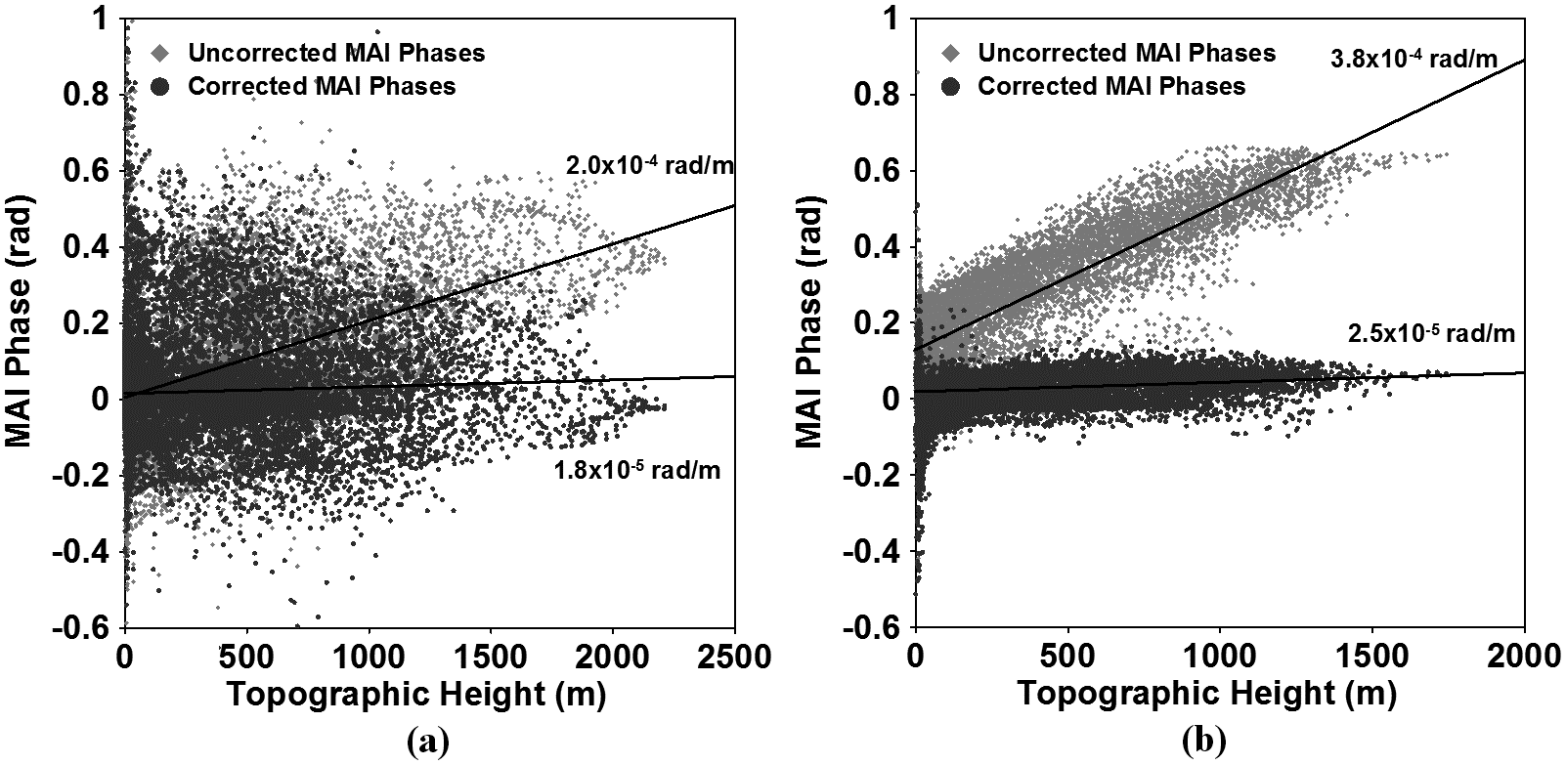
Variation of MAI phases in the (a) ascending and (b) descending acquisition pairs with respect to topographic height before and after the topographic correction. The bright gray solid diamonds and dark gray solid circles indicate the uncorrected and corrected MAI phase, respectively.

**Fig 4:**
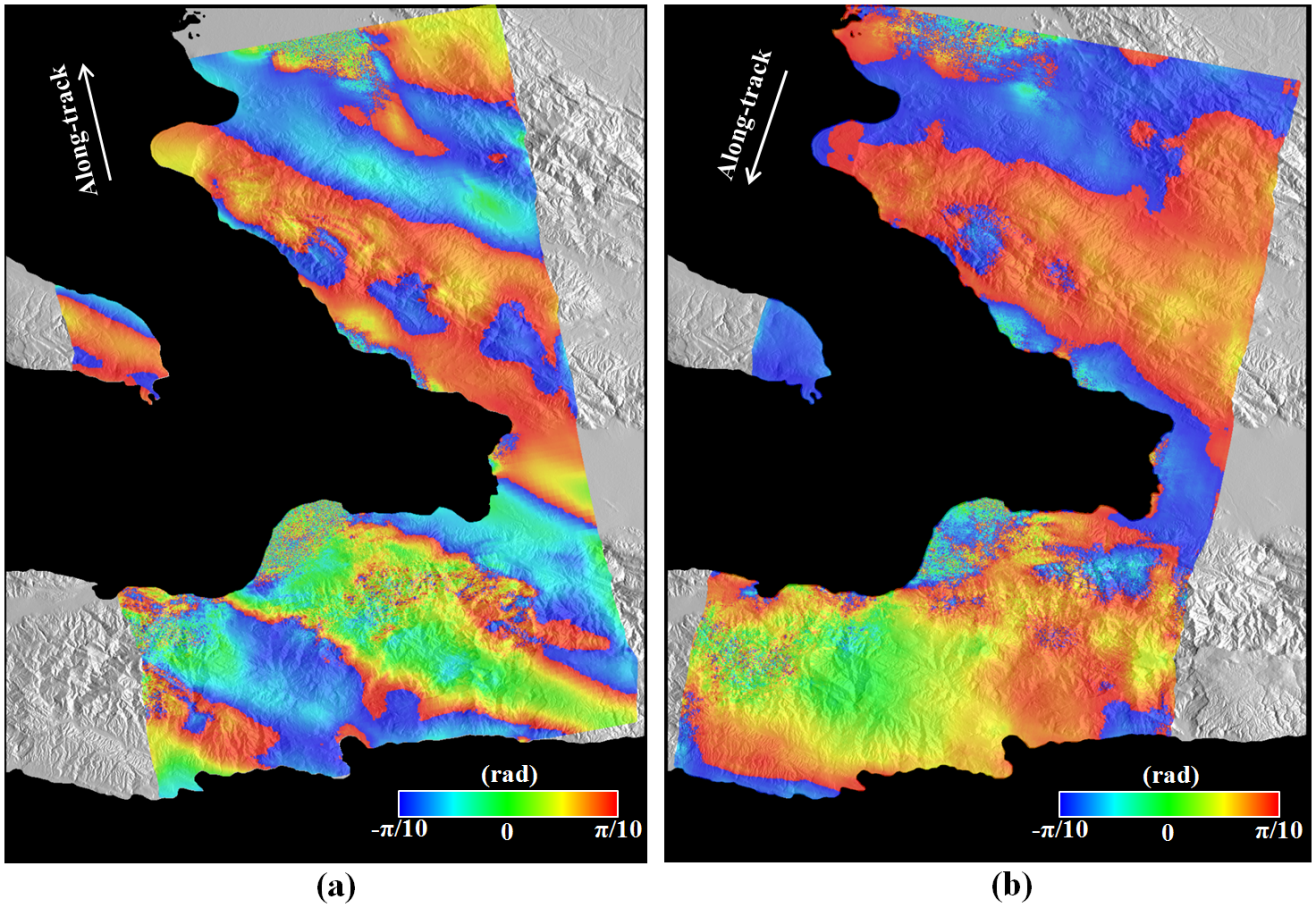
MAI interfeograms after correcting topographic distortions from (a) ascending and (b) descending interferometric pairs. It is noted that the ascending MAI interferogram possesses severe ionospheric distortion.

Fig 2(c) and 2(d) show the MAI interferograms generated from the ascending and descending pairs, respectively. The MAI interferograms have been created by the MAI approach improved by [18,19]. The MAI processing of the ascending pair has been performed by using the forward, average and backward Doppler centroids of 480.6, 76.5 and -327.5 Hz and the sub-aperture processing bandwidth of 801.7 Hz. The descending MAI processing has been carried out from the forward, average and backward Doppler centroids of 333.4, -70.7 Hz and -474.7 Hz and the sub-aperture processing bandwidth of 794.8 Hz. The normalized squint of 0.5 and the effective antenna length of 8.9 m have been imposed on the ascending and descending MAI calculations. Forward- and backward-looking SLC images have been created from ALOS PALSAR raw signal data, and then the forward- and backward-looking interferograms for the ascending and descending acquisitions have been generated and multilooked by using the two-step strategy: 2x4 and 4x4 multi-look processing in the range and azimuth. The multilooked MAI interferograms have been smoothed by the Goldstein filter used for the InSAR processing. The used filter size was as large as 64 because the interferometric coherence of the inteferometric pairs used for this study is low. Finally, the ascending and descending MAI interferograms have been generated after residual flat-Earth and topographic phase corrections are applied by using the perpendicular baseline difference [18,19]. The detailed MAI parameters are summarized in Table 1. Although the topographic phase distortions caused by the perpendicular baseline difference were corrected by the improved MAI processing [18], the severe topographic distortions still remained in the ascending and descending MAI interferograms, as shown in Fig 2(c) and 2(d). The distortion is due to a mis-registration between the forward- and backward-looking interferograms. The forward-looking interferogram has slightly different viewing angle from the backward-looking interferogram, and hence the forward- and backward-looking interferograms have a different geometric distortion that is proportional to the topographic height. The geometric difference causes the topographic phase distortion in the MAI inteferograms. Fig 3 shows the variation of the MAI phases in the ascending and descending pairs with respect to topographic height. The bright gray solid diamonds denote the uncorrected MAI phase and the dark gray solid circles indicate the corrected MAI phase. It is very clear that the uncorrected MAI phase is linearly correlated with topographic height in both the pairs in Fig 3(a) and 3(b). Thus, the topography-distorted MAI phase was easily corrected by a linear regression method. The slopes estimated by the regression analysis were about 2.0x10-4 and 3.8x10-4 rad/m for the ascending and descending pairs, respectively. The slope of the descending MAI phase is almost two times larger than that of ascending MAI phase because the perpendicular baseline of the descending MAI phase is larger than the ascending MAI phase. After the slopes were corrected, the MAI phase variations with respective to the topographic height were mitigated as seen in the bright gray solid diamonds of Fig 3(a) and 3(b). In addition, the ascending MAI phase is more scattered than the descending MAI phase. This is because the ascending MAI interferogram has lower coherence as well as severe ionospheric distortion. The severe ionospheric effect can be further identified in Fig 4(a).

**Fig 5:**
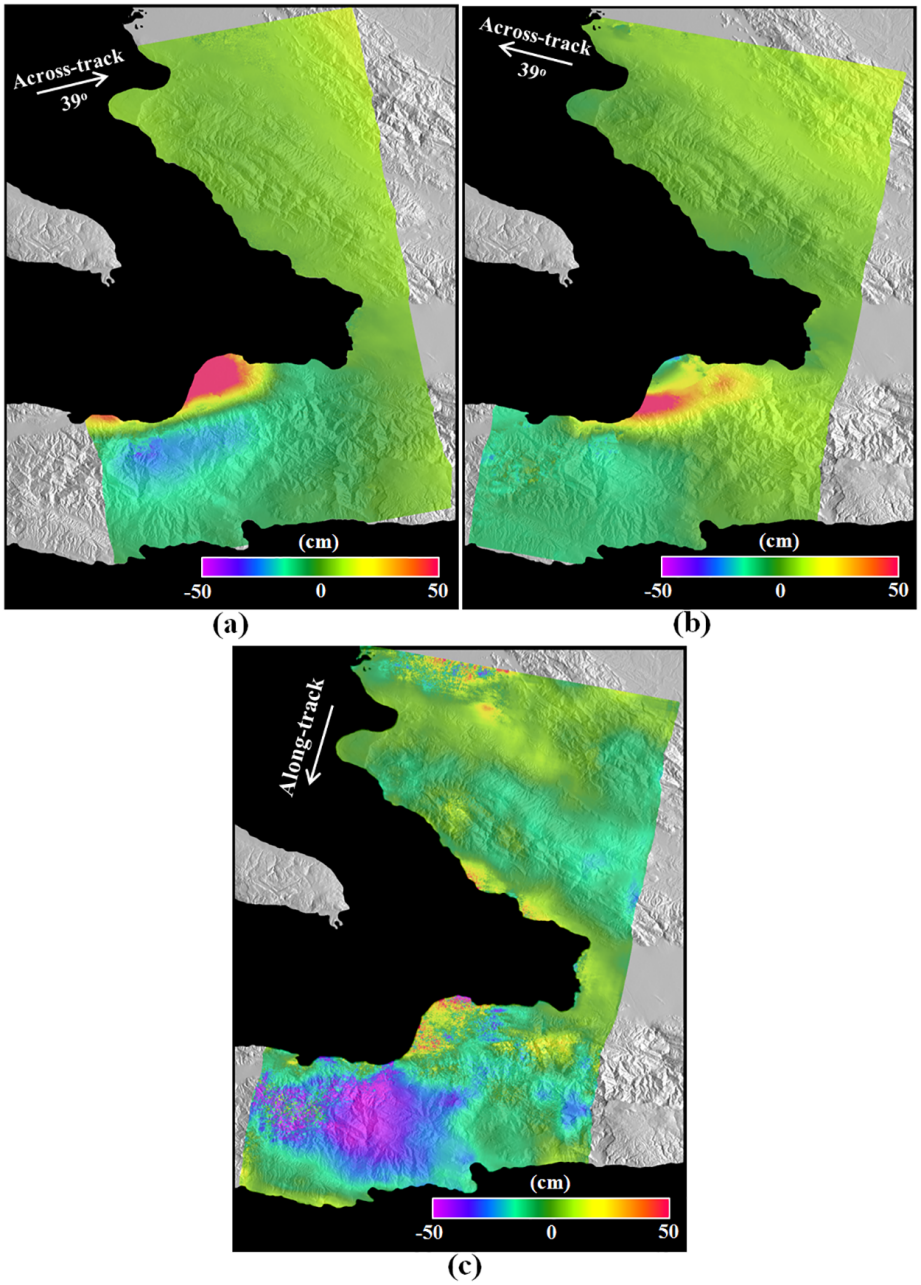
(a,b) LOS surface deformations measured by the InSAR technique from the ascending and descending pairs (see Fig 2(a) and 2(b)), and (c) along-track surface deformation measured by the MAI method from the descending pair (see Fig 3(b)). The ascending MAI interferogram could not be used to measure the along-track deformation due to the severe ionospheric distortion as shown in Fig 3(a).

Fig 4 shows the MAI interferograms after the additional topographic correction. As aforementioned, the ascending MAI interferograms of Fig 4(a) shows a prominent “azimuth streak” pattern, which is a well-known effect caused by ionospheric distortion. The ionospheric phase reaches from about -0.15π to 0.15π rad., which correspond to the surface deformations from about -67 to 67 cm. Although the ionospheric distortion can be reduced by the directional filtering [34], an effective correction of the ionospheric distortion is very difficult because the deformation and ionosphere signals are mixed. Thus, we didn’t use the ascending MAI interferogram when 3D surface deformation was retrieved, because the ascending MAI interferogram can work as noise. Fig 5 shows the LOS surface deformations converted from the ascending and descending InSAR interferograms and the along-track surface deformation from the descending MAI interferogram. Supposed that the ascending and descending LOS deformations are similar, it indicates that upward or downward deformations are dominant, while if the two LOS deformations are different, it means that horizontal deformations are dominant.

**Fig 6:**
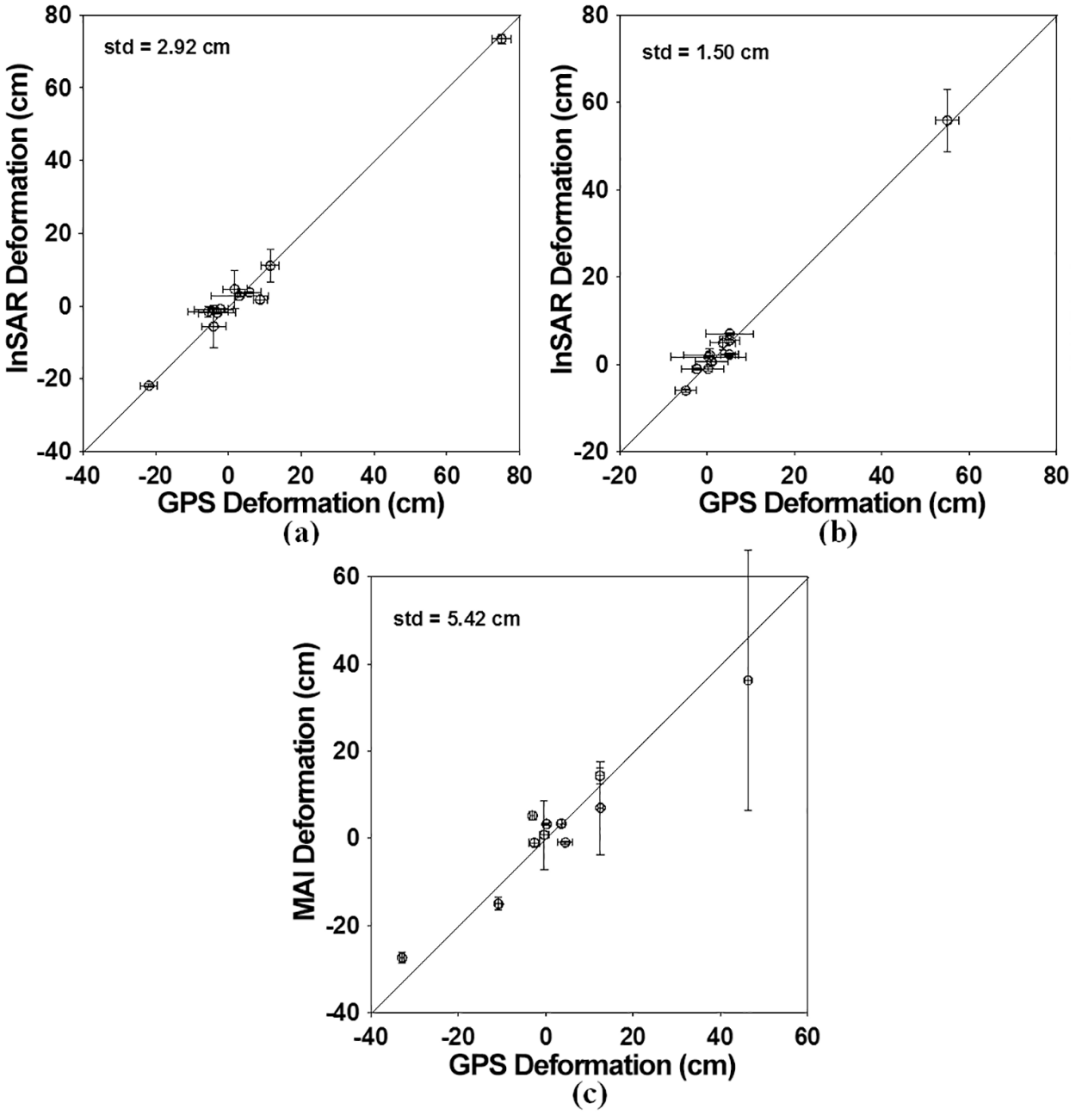
Comparison between InSAR/MAI and GPS deformation measurements: (a,b) ascending and descending InSAR verse GPS measurements and (c) descending MAI verse GPS measurements. The GPS deformations have been measured by [25]. The bars denote uncertainties of the InSAR/MAI and GPS measurements at two standard deviations. The standard deviations of the InSAR and MAI measurements were estimated by using the window kernel of 5x5.

However, it is certainly not easy to understand the 3D deformation field from the LOS imaging geometries. Since the 3D deformation enables us to easily understand the deformation patterns, the 3D deformation field needs to be retrieved. The along-track surface deformation presented in Fig 5(c) indicates the horizontal surface deformation in the orbit-track direction. Positive deformations mean deformations in the along-track direction (approximately South direction), while negative deformations are deformations in the opposite direction of the along-track (approximately North direction). We can see the positive deformation in the city of Leogane while the negative deformation in the city of Jacmel (Fig 5(c)). Fig 6(a) and 6(b) compare the InSAR LOS deformations with the GPS LOS deformations. The GPS LOS deformations were measured from the GPS stations and projected into the InSAR geometries. Fig 6(c) compares the along-track deformations from MAI with the GPS along-track deformations projected into the MAI geometry. The archived standard deviations were about 2.92, 1.50 and 5.42 cm in the ascending and descending InSAR and MAI measurements, respectively. The descending InSAR measurement was two times better than the ascending InSAR measurement. It is because the coherence of the descending pair is higher than that of the ascending pair and the ascending pair includes the severe ionospheric phase distortion. The achieved accuracy of the MAI measurement was approximately two to four times larger than the InSAR measurements. As aforementioned, one fringe is about 11.8 cm for the InSAR measurements while about 890 cm for the MAI measurement. It may be expected that MAI accuracy is about 75 times worse than InSAR accuracy. However, because InSAR measurements include the tropospheric artifacts while MAI measurements do not depend on the tropospheric artifacts, it was reported that the accuracy of MAI measurements is two times worse than that of InSAR measurements [2,6]. The MAI measurement in this study was worse than the normal case due to high topographic distortion and low interferometric coherence.

**Fig 7:**
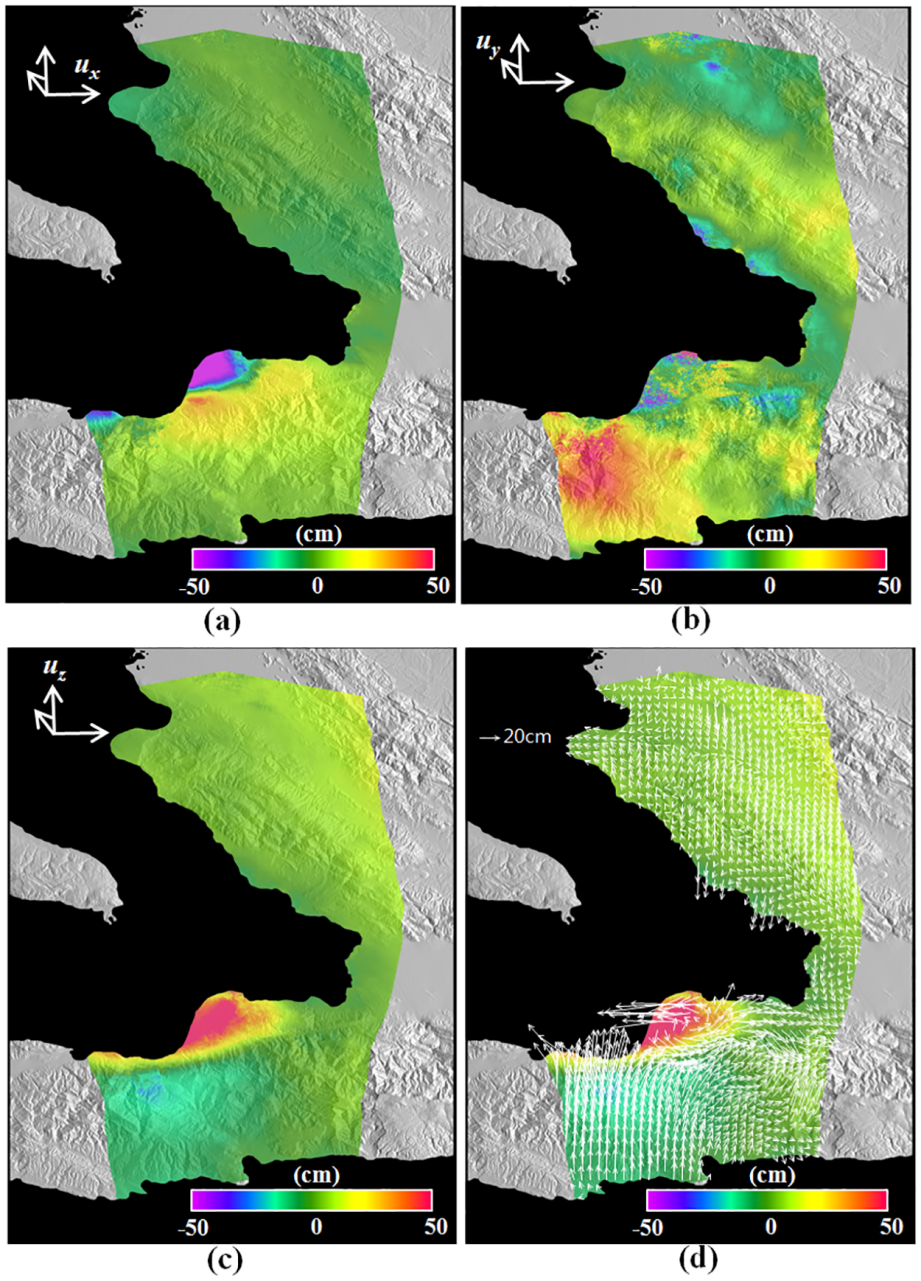
Three-dimensional surface deformation field for the 2010 Haiti earthquake retrieved from the LOS and along-track deformations of Fig 5. (a) East (*u*_*x*_), (b) north (*u*_*y*_), and (c) up (*u*_*z*_) deformations constructed by integrating the InSAR and MAI displacement maps. (d) Three-dimensional deformation map displayed by using arrows and color maps. The arrows represent the horizontal deformation vectors and the colors denote the vertical deformations.

Fig 7 represents the 3D surface deformation field retrieved from the ascending and descending LOS deformations and descending along-track deformation shown in Fig 5. The 3D retrieval was performed by using Eq 8, and the weighting matrix in Eq 8 was generated by using the standard deviations of the InSAR and MAI measurements, which were calculated from the GPS in-situ measurements. For a possible improvement of the 3D retrieval, an InSAR noise covariance matrix can be considered to mitigate the impact of spatial correlated nature of InSAR and MAI observations [35]. From Fig 7(a), the eastward horizontal deformation of more than 50 cm in the city of Leogane was measured and the westward horizontal deformation of on average 30 cm was observed in the mountainous area between the two cities of Leogane and Jacmel. It is clear that the horizontal surface deformation in the east direction shows a behavior of strike-slip fault. However, the northward horizontal deformation of about 20 to 50 cm was very clear in the left side of Leogane and Jacmel as seen Fig 7(b). Most of all, the southward horizontal deformation was observed in the city of Leogane. It can be understood that the NS deformation is a normal fault. Moreover, we were able to find the clear uplift in the city of Leogane, while the subsidence of about 10 to 20 cm occurred in the mountainous area between the two cities of Leogane and Jacmel as seen Fig 7(c). This upward deformation further matches the normal fault pattern. This deformation pattern cannot be understood by using a simple fault model. This is the very reason why a single fault made of two subsegments [27] and a more complex geometry with multiple faults [28] have been proposed. Fig 7(d) shows the 2010 Haiti 3D deformation map that is displayed by using arrows and color maps. The arrows represent the horizontal deformation vectors and the colors denote the vertical deformations. We can see from the horizontal vectors both the north-south and east-west mixed deformations and the up-down deformations in city of Leogane.

**Fig 8:**
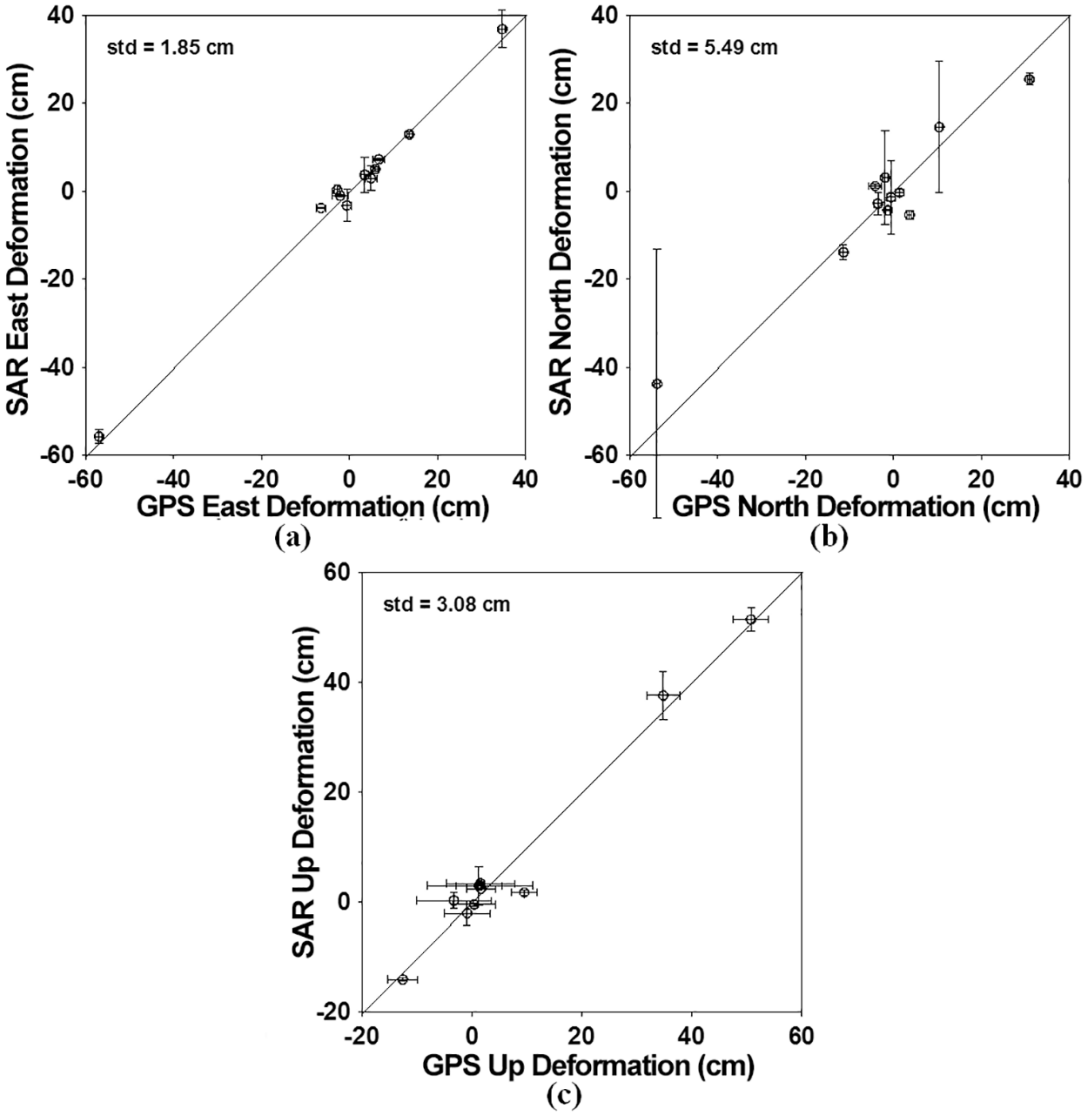
Comparison of the 3D deformations estimated by integrating the InSAR and MAI methods with the 3D deformations from GPS measurements: (a) east, (b) north and (c) up components. The bars denote the uncertainties of SAR and GPS measurements at two standard deviations. The standard deviations of the SAR measurements were estimated by using the window kernel of 5x5.

Fig 8 compares between the SAR-derived 3D deformation and the GPS-derived 3D deformations. The ascending and descending InSAR measurements are dominant in the calculation of the east and up deformations, while the MAI measurement is dominant in the north deformation calculation. Thus, the north deformation accuracy of about 5.49 cm was more than two times lower than the east deformation accuracy of about 1.85 cm and the up deformation accuracy of about 3.08 cm. The up deformation accuracy was much lower than the east deformation accuracy. This is because the accuracy of the GPS measurement in the up direction is much lower than horizontal direction.

**Fig 9:**
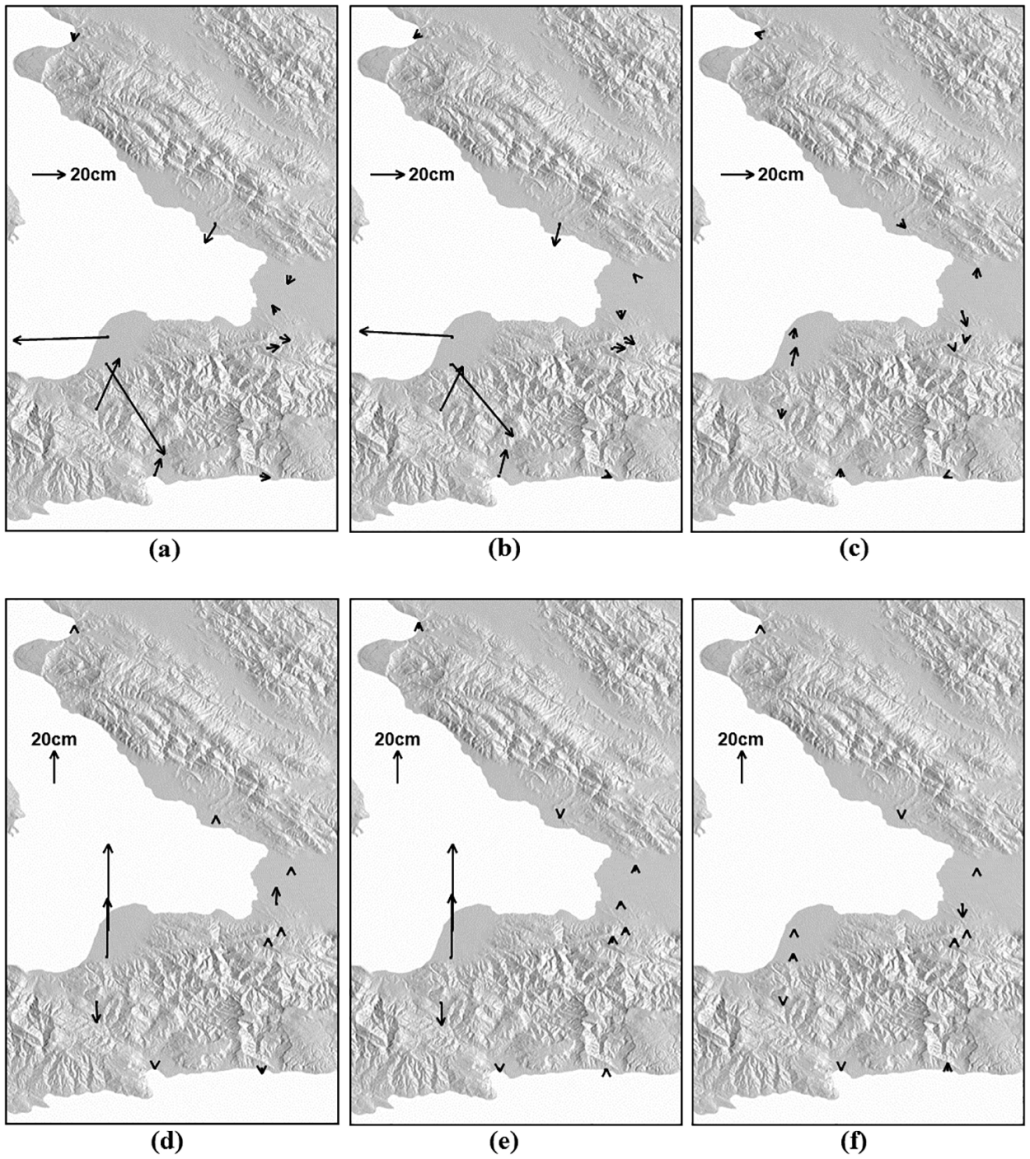
Spatial comparison between GPS and SAR measurements: Horizontal and vertical deformations of (a,d) GPS, (b,e) SAR and (c,f) residuals.

The spatial comparison between GPS-derived and SAR-derived 3D deformations was performed as seen in Fig 9. Fig 9(a), 9(b) and 9(c) show the horizontal deformations in the GPS and SAR measurements and the residuals between GPS and SAR, respectively, and Fig 9(d), 9(e) and 9(f) respectively present the up deformations in the GPS, SAR and residual. Fig 9 shows that the deformation patterns between the GPS and SAR measurements were well matched. However, the residuals in the city of Port-au-Prince and the mountainous area near the city of Leogane were relatively larger than other sites as seen Fig 9(c). Since the large residual vectors were northward or southward, deformation errors are dominant in the north direction. It indicates that the MAI measurement has lower accuracy. The InSAR measurement of Fig 9(d) in Port-au-Prince was larger than the GPS measurement of Fig 9(e). This would be 1) because GPS errors in the up direction are relatively large and a GPS measurement just presents a deformation in at the GPS station or 2) because the atmospheric artifact or DEM error can happen when the InSAR pairs were processed. The 3D surface deformation field retrieved by the InSAR and MAI combination showed behavior of strike-slip fault in the east direction as well as a behavior of normal fault in the north-south direction. The measured 3D deformation field gave us to a better understanding of the fault’s behavior of the 2010 Haiti earthquake. If 3D deformations of an earthquake can be retrieved by integrating the InSAR method with the MAI method, it would be much easier to understand the fault’s behavior.

## Conclusions

For sustainable advances in the geological interpretation of geohazards such as earthquakes and volcanic eruptions, 3D surface deformation mapping has been believed to be more and more essential. The SAR systems made possible the precise measurement of the 3D surface deformation field by using the InSAR and MAI combination. In this paper, we three-dimensionally observed the surface deformation caused by the 2010 Haiti earthquake. For the measurement, we used the ALOS PALSAR FBS ascending pair, which was acquired on February 9, 2008 and February 14, 2010, and the descending pair, which was on March 9, 2009 and January 25, 2010. Two InSAR and two MAI interferograms were generated from the ascending and descending pairs. Albeit their interferometric coherences were relatively low, the LOS and along-track deformations could be measured from the InSAR and MAI interfrograms. Most of all, it was very difficult to measure the along-track deformation, because the MAI interferograms had severe topographic distortions. However, the distortions could be successfully mitigated by the additional topographic corrections. Nevertheless, one of the two MAI interferograms had a severe ionospheric distortion, and hence we could not use the ionosphere-distorted MAI interferogram for retrieving 3D deformations.

The LOS and along-track surface deformations were calculated from the InSAR and MAI interferograms. We could see clear deformations in the city of Leogane and the mountainous area between the cities of Leogane and Jacmel (Fig 7). When compared with the GPS in-situ measurements, the archived standard deviations were about 2.92 and 1.50 cm in the ascending and descending LOS deformations and about 5.42 cm in the descending along-track deformations, respectively. Because the coherence of the descending pair is higher than that of the ascending pair and the ascending pair includes the severe ionospheric phase distortion, the descending LOS deformation accuracy was much higher. The 3D deformation field was retrieved by the combination of the ascending and descending LOS deformations and the descending along-track deformation. The retrieved 3D deformation field simultaneously presents a behavior of the left-lateral strike-slip fault in the east deformation and a behavior of the normal fault in the north and up deformations (Fig 7). The behavior of the strike-slip fault was shown in the eastward horizontal deformation of more than 50 cm in the city of Leogane as well as the westward horizontal deformation of on average 30 cm in the mountainous area between the two cities of Leogane and Jacmel. Moreover, the behavior of the normal fault was seen in the southward horizontal deformation in the city of Leogane as well as the northward horizontal deformation of about 20 to 50 cm in the left side of Leogane and Jacmel. It indicates that the deformation pattern cannot be understood by using a simple fault model. When compared to the GPS in-situ measurements, the achieved measurement accuracy was about 5.49, 1.85 and 3.08 cm in the east, north and up deformations, respectively. The north accuracy was much worse than the east and north accuracies because the measurement in the north direction was calculated from the MAI measurement. The spatial pattern of the SAR measurement errors was analyzed. It further confirms that the deformation patterns between the GPS and SAR measurements were well matched. The 3D surface deformation field retrieved by the InSAR and MAI combination showed a behavior of strike-slip fault in the east direction as well as a behavior of normal fault in the north-south direction. The fault’s behavior of the 2010 Haiti earthquake will be very vital to provide important insights into a geological process of the earthquake.
